# Analysis of Ophthalmic Artery Doppler in Normotensive, Preeclamptic, and Eclamptic Pregnancies in Correlation With Clinical Parameters in a Tertiary Care Hospital in India

**DOI:** 10.7759/cureus.74696

**Published:** 2024-11-28

**Authors:** Guru Yogendra Muthyal, Anil Kumar Sakalecha, Goravimakalahalli Srinivasareddy Hemanth Kumar, Shantala Sawkar, Vamsi Venkat

**Affiliations:** 1 Radiodiagnosis, Sri Devaraj Urs Medical College, Tamaka, IND; 2 Radiology, Sri Devaraj Urs Medical College, Tamaka, IND

**Keywords:** creatinine, eclampsia, hypertension, platelets, pregnancy

## Abstract

Abstract

Preeclampsia occurs in hypertensive pregnant women beyond 20 weeks of gestation and is accompanied by proteinuria. Hypertensive retinopathy is the most prevalent sign of preeclampsia, and eclampsia and it needs to be addressed at the earliest opportunity. This study was intended to gauge and assess the ophthalmic artery Doppler indices such as mean enveloped velocity, the pulsatility index (PI), and the resistivity index (RI) in normotensive, preeclamptic, and eclamptic pregnancies with their respective correlations. We also saw correlations with serum creatinine and platelets with the PI and RI.

Methods

A prospective case-control study was conducted among 70 subjects in the Department of Radiodiagnosis of Sri Devaraj Urs Medical College, Tamaka, Karnataka, India. We carried out ultrasound and Doppler examinations of the right ophthalmic artery. Doppler indices including mean enveloped velocity, PI, and RI were assessed in normotensive as well as preeclamptic pregnancies.

Results

The mean ages of cases and controls were 28.26 and 26.74 years, respectively. The mean RIs in controls and cases were 0.86 and 0.69, respectively (preeclampsia and eclampsia were 0.73 and 0.65, respectively), whereas the mean PIs in controls and cases were 1.96 and 1.17, respectively (preeclampsia and eclampsia were 1.31 and 1.06, respectively). The mean enveloped velocities in preeclampsia and eclampsia were 28.82 cm/sec and 20.43 cm/sec, respectively.

Conclusion

We found a definite reduction in the PI, RI, and mean enveloped velocity flow levels in preeclampsia and eclampsia patients in comparison to that amongst normal subjects. Therefore, it is important to assess and manage such conditions at the earliest stage to achieve a better prognosis while providing adequate treatment to the patient.

## Introduction

Preeclampsia is a multisystem disease that occurs in 5-10% of pregnancies in patients who were previously normotensive and were diagnosed with hypertension beyond 20 weeks of gestation and is accompanied by proteinuria. It is more common in primigravida women with, in severe cases, organ damage to the liver, kidney, heart, lungs, and brain [1-5]. The most common risk factors for preeclampsia are primigravida, a family history of preeclampsia, chronic renal disease, chronic hypertension, a previous preeclamptic history, a high BMI, obesity, diabetes mellitus, anti-phospholipid syndrome, twin gestation, extreme age (≥ 40 years), Black race, and the angiotensinogen gene [3-5]. Preeclampsia, which resolves six weeks postpartum, is defined as increased blood pressure > 140/90 mmHg recorded on two consecutive occasions at least six hours apart along with the presence of proteinuria beyond a gestational age of 20 weeks in a hitherto normotensive and non-proteinuric woman [3-5].

Approximately 30-100% of preeclampsia patients suffer from eye problems [3]. Accordingly, hypertensive retinopathy is the most prevalent sign of preeclampsia and eclampsia, affecting 60% of patients, and among these, the severity of ocular abnormalities varies with the level of severity of preeclampsia in a proportionate sequence [3,6]. Visual disturbance occurs in 25% of women experiencing severe preeclampsia, although blindness occurs rarely at a rate of 1-3% in eclampsia patients [3,6]. Visual symptoms of preeclampsia and eclampsia encompass visual field abnormalities, photopsia, hazy or reduced vision, and full blindness (severe stage) [3,6]. The three most prevalent eye problems are exudative retinal detachment, hypertensive retinopathy, and cortical blindness [3].

In severe cases, the arrival of blindness in such patients is commonly caused by retinal abnormalities such as edema and vascular alterations including retinal arteriolar vasospasm, central retinal artery thrombosis, and retinal detachment. Transient cortical impairment of vision owing to focal cerebral edema is thought to occur in 1-15% of eclamptic women [7]. Such retinal alterations are likely to develop when diastolic blood pressure > 100 mmHg and systolic blood pressure > 150 mmHg [3].

Transcranial Doppler ultrasound is a safe and fast method to study intracranial vessels, yet it is challenging to use, offers poor spatial resolution, and requires substantial technical expertise for accuracy [8,9]. Additionally, transcranial Doppler imaging equipment is often unavailable in many low-income nations. Conversely, ophthalmic artery Doppler is economical, accurate, repeatable, and noninvasive and does not subject the patient to ionizing radiation exposure. Inspecting the ophthalmic artery is technically feasible because eyeballs lack bone, fat, or gas structures. This technique also boasts a predictive value for developing early-onset preeclampsia similar to uterine artery Doppler evaluation [8,10].

Because of limited comprehension of the function of the ophthalmic artery, Doppler alterations and hemodynamic abnormalities associated with preeclampsia, eclampsia, and its consequences are seen [10,11]. We assessed the ophthalmic artery Doppler indices (mean enveloped velocity flow, PI, RI) in normotensive and preeclampsia pregnancies and the association of the Doppler indices with artery Doppler and other clinical parameters [12,13].

## Materials and methods

This was a prospective case-control study conducted among 70 (35 cases and 35 controls) subjects in the Department of Radiodiagnosis of Sri Devaraj Urs Medical College, Tamaka, Karnataka, India, in 2024. This study was submitted to and approved by the Central Ethics Committee of Sri Devaraj Urs Academy of Higher Education and Research (approval number: SDUAHER/KLR/R&D/CEC/S/PG/55/2024-25). Written informed consent was obtained from the subjects after explaining in detail the objective of the study and providing the assurance that their refusal would not influence the quality of their care.

Inclusion and exclusion criteria

All pregnant women diagnosed with preeclampsia and eclampsia with a gestational age > 20 weeks and with blood pressure (BP) ≥ 140/90 mmHg, proteinuria exceeding 0.3 g/l in a 24-hour urine sample, and at least a 2+ on a dip-stick random urine test were included. A diagnosis of eclampsia required a gestational age of > 20 weeks with a sustained BP equal to or above 160/110 mmHg. Women diagnosed with diabetes, on corticosteroids, with a history of addiction, ocular disease, vasculitis, or other vascular diseases, and who did not provide consent were excluded from the study.

Procedure

**Figure 1:**
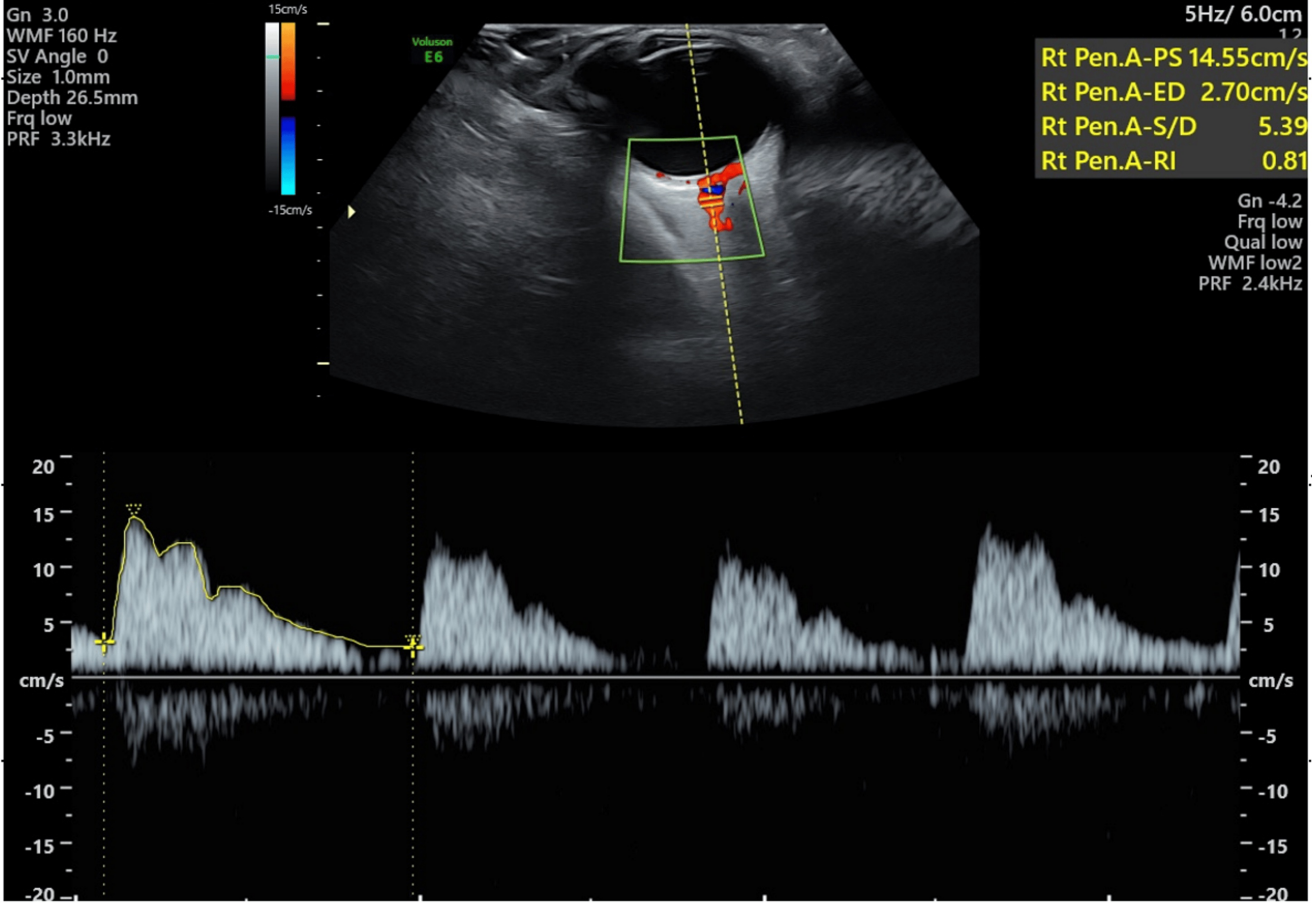
Doppler imaging of the right ophthalmic artery showing mean enveloped velocity and Resistivity Index (RI).

**Figure 2:**
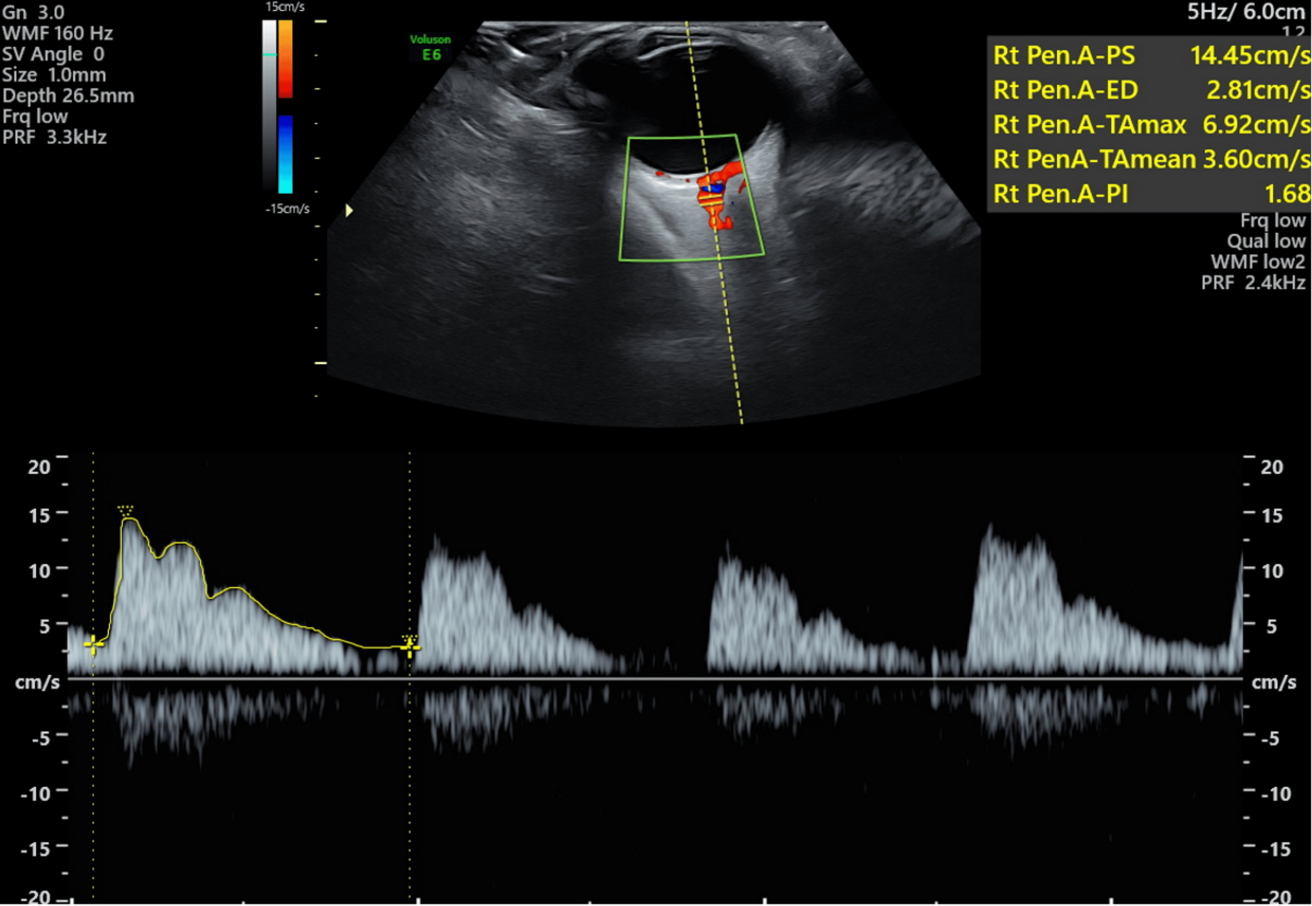
Doppler imaging of the ophthalmic artery showing mean enveloped velocity and Pulsatility Index (PI).

All 70 subjects were subjected to ophthalmic examination in the form of ocular ultrasonography executed by an experienced radiologist within 24 hours of admission. We carried out ultrasound and Doppler examinations of the right ophthalmic artery. Doppler indices including mean enveloped velocity flow, PI, and RI were assessed in normotensive as well as preeclamptic and eclamptic pregnancies. Both clinical and laboratory findings were recorded and correlated with other Doppler parameters as shown in Figure 1 and Figure 2. RI and PI were also correlated with serum creatinine and platelets.

Sample size calculation

The sample size was projected using the difference in mean RIs between cases and controls in the study by Olatunji et al. as 0.62 ± 0.1 and 0.7 ± 0.1. At a 95% confidence limit (CI) and 80% power, 32 samples in each group were obtained with a 10% nonresponse, accounting for a sample size of 32 + 3.2 ≈ 35 cases. The sample size was estimated using the formula $N=2 SD^{2}\frac{\left ( Z\alpha/2 +Z\beta\right )^{2}}{d^{2}}$, where Zα=1.96 for an error of 5%, Zβ=0.84 for a power of 80%, SD is standard deviation, d is difference.

Data analysis

Data were entered into a Microsoft Excel data sheet (Microsoft Corporation, Redmond, Washington, United States) and analyzed using IBM SPSS Statistics for Windows, Version 22.0 (Released 2016; IBM, Armonk, New York, United States). Categorical data will be represented in the form of frequencies and proportions. Continuous data were denoted as mean and standard deviation (SD). An independent t-test was used as the test of significance to detect the mean differences. P values < 0.05 were considered statistically significant.

## Results

The mean age of cases and controls was found to be 28.26 ± 4.52 years and 26.74 ± 3.18 years, respectively, which was statistically significant (p < 0.05). The mean RIs in controls and cases were 0.86 and 0.69, respectively (preeclampsia and eclampsia were 0.73 and 0.65, respectively), and the mean RI was statistically significant (p < 0.05), as shown in Table 1. 

**Table 1:** Table showing resistivity index in controls, preeclampsia, and eclampsia groups. *The result is significant at p < 0.05

Resistivity index	Control group (n=25)	Preeclampsia Group (n=16)	Eclampsia Group (n=19)	P value
Mean ± SD	0.86 ± 0.06	0.73 ± 0.07	0.65 ± 0.07	< 0.0001*

The mean RIs in cases and controls with platelets >150 x 10^9^/L were 0.67 and 0.86, respectively, which was statistically significant (p < 0.05). The mean RI in cases with < 150 x 10^9^/L platelets was 0.69 ± 0.08, as shown in Table 2.

**Table 2:** Correlation of platelets with resistivity index (RI) in cases and controls. *The result is significant at p < 0.05; ** Cases: preeclampsia and eclampsia patients.

Platelets	RI in cases**, mean ± SD	RI in controls, mean ± SD	P value
< 150 x 10^9^ mg/dL	0.69 ± 0.08	-	-
> 150 x 10^9^ mg/dL	0.672 ± 0.09	0.86 ± 0.06	< .00001

The mean RIs in cases and controls with serum creatinine of 0.6-1.1 mg/dL were 0.73 and 0.86, respectively, which was statistically significant (p < 0.05). The mean RIs in cases and controls with a serum creatinine of ≥1.2 mg/dL were 0.68 and 0.86, which was statistically significant (p < 0.05), as shown in Table 3. 

**Table 3:** Correlation of serum creatinine with resistivity index. * The result is significant at p < 0.05; **Cases: preeclampsia and eclampsia patients RI: Resistivity Index

Serum creatinine	RI in cases**, mean ± SD	RI in controls, mean ± SD	P value
0.6-1.1 mg/dL	0.732 ± 0.08	0.86 ± 0.06	0.000035*
≥1.2 mg/dL	0.68 ± 0.08	0.86 ± 0.0	0.000013*

The mean PIs in controls and cases were 1.96 and 1.17, respectively (preeclampsia and eclampsia were 1.31 and 1.06, respectively), which was statistically significant, as shown in Table 4. 

**Table 4:** Mean pulsatility index in controls and cases (preeclampsia and eclampsia). *The result is significant at p < 0.05

Pulsatility index	Controls (n=35)	Preeclampsia (n=16)	Eclampsia (n=19)	P value
Mean ± SD	1.96 ± 0.29	1.31 ± 0.21	1.06 ± 0.18	< 0.0001*

The mean PIs in cases and controls with platelets >150 x 10^9^/L were 1.14 and 1.38, respectively, which was statistically significant (p < 0.05). The mean PIs in cases with < 150 x 10^9^/L platelets were 1.14 ± 0.22, respectively, as shown in Table 5.

**Table 5:** Correlation of platelets with pulsatility index in cases and controls. *The result is significant at p < 0.05; **Cases: preeclampsia and eclampsia patients PI: Pulsatility Index

Platelets	PI in cases**, mean ± SD	PI in controls, mean ± SD	P value
< 150 10^9^ mg/dL	1.14 ± 0.22	-	-
> 150 10^9^ mg/dL	1.38 ± 0.18	1.96 ± 0.29	0 .000051.*

The mean PIs in cases and controls with a serum creatinine of 0.6-1.1 mg/dL were 1.28 and 1.97, respectively, which was statistically significant (p < 0.05). Similarly, the mean PIs in cases and controls with a serum creatinine of ≥1.2 mg/dL were 1.16 and 1.9, respectively, which was statistically significant (p < 0.05), as shown in Table 6. 

**Table 6:** Correlation of serum creatinine and pulsatility index in cases and controls. * The result is significant at p < 0.05; **Cases: preeclampsia and eclampsia patients RI: Resistivity Index.

Serum creatinine	PI in cases**, mean ± SD	PI in controls, mean ± SD	P value
0.6-1.1 mg/dL	1.28 ± 0.23	1.97 ± 0.30	< 0.00001*
≥1.2 mg/dL	1.16 ± 0.23	1.9 ± 0.0	< 0.00001*

The mean enveloped velocities in these patients wherein we found the mean enveloped velocities in preeclampsia and eclampsia were 28.82 cm/seconds and 20.43 cm/seconds, respectively, which was also statistically significant (p < 0.05), as shown in Table 7.

**Table 7:** Enveloped velocity in preeclampsia and eclampsia. *The result is significant at p < 0.05

Mean enveloped velocity flow in cm/sec	Preeclampsia (n=17)	Eclampsia (n=19)	P value
Mean ± SD	28.82 ± 3.59 cm/sec	20.43 ± 4.09 cm/sec	0.042*

## Discussion

Hypertension is not an uncommon disorder today. The change in lifestyle compared to earlier times has played a role in the exponential increase in the incidence of several cases of hypertension. Hypertension may not be detected as a serious problem in the early stages but has been found to impact the individual significantly. During pregnancy, hypertensive disorders are classified into five types: eclampsia, gestational hypertension, chronic hypertension (essential or secondary), preeclampsia, and preeclampsia overlaid upon chronic hypertension [8].

Preeclampsia, the most common medical complication of pregnancy, is characterized by elevated systemic blood pressure (> 140/100 mmHg), proteinuria (> 300 mg/24 hours), and generalized edema, and it typically appears after the 20th week of pregnancy. It occurs in 5-10% of all pregnancies [14]. Severe preeclampsia presents with systemic blood pressure over 160/110 mmHg, proteinuria 2 g/24 hours, serum creatinine > 2 mg/dL, oliguria, thrombocytopenia, epigastric pain, cerebral and visual disruptions, headache, pulmonary edema, and elevated liver enzymes. In addition to the symptoms of preeclampsia, convulsions before or after birth indicate progression to eclampsia [14]. 

The most common ophthalmologic presentation of preeclampsia is vasoconstriction of the retinal arterioles, the incidence of which rises with severity. The condition is typically asymptomatic and resolves without causing any complications after delivery. Other clinically significant manifestations are extremely rare including cortical blindness and retinal serous detachment. The link between the two disorders is unusual [7,15].

Preeclampsia or eclampsia can cause retinal and choroidal circulation abnormalities as well as other fundoscopic symptoms including vision loss. Such patients may develop severe hypertensive retinopathy, which includes retinal hemorrhage, subretinal serous fluid buildup, papilledema, and Elschnig spots. Other common visual problems related to preeclampsia include Purtscher-like retinopathy, serous retinal detachment, cortical blindness, retinal or vitreous hemorrhages, and central retinal vein occlusions. Visual warning signs can consist of double or blurred vision, abrupt transitory vision loss, and flashing lights as well as visual field grievances such as homonymous hemianopia. Cortical blindness is a severe form of vision loss in preeclampsia. The unexpected loss of eyesight is a rare consequence of severe preeclampsia [14,16,17].

The ophthalmic artery provides easy access to assess maternal cardiovascular changes. The ocular artery with its functional, embryological, and anatomical parallels to intracranial vessels can provide insights into the small-caliber intracerebral vasculature and its hemodynamics, which are difficult to scan transcranially [8].

Sahana et al. reported the mean RIs of preeclampsia cases and controls as 0.64 and 0.78, respectively [18]. Also, Kumari et al. reported the mean RIs of normotensive and preeclampsia participants in their study were 1.0 and 1.0 (0.95 on the left and 1.05 on the right), respectively (p < 0.05) [8]. Similar to our study, Madina et al. reported the mean RIs of preeclampsia cases and controls to be 0.63 and 0.71 [19], which was also confirmed by de Oliveira et al., who reported an RI of 0.75 in normotensive pregnant women and 0.63 in preeclamptic women [20]. In the current study, we found a clear drop in the mean RI levels in eclampsia levels compared to preeclampsia patients, which was even more evident when assessed against controls. Our results are in accordance with those of Olatunji et al., who found the mean RIs in cases and controls to be 0.62 and 0.71, respectively [21]. Therefore, a definite relationship exists between preeclamptic and eclamptic patients regarding the ophthalmic artery, which was clearly established in the changes in the mean RI levels in comparison to that of normal subjects.

Hypertension also has profound effects on other organ systems in the body. Therefore, we assessed the platelets as well as serum creatinine levels in hypertensive patients and correlated them with RI levels, and we found that the mean RIs in cases and controls with platelets >150 x 10^9^/L were 0.67 and 0.86, respectively, which was statistically significant. A correlative assessment of platelets against the RI levels revealed a drop in the mean RI levels in cases when compared to controls. Whether platelets can be used to detect changes in the RI of the ophthalmic artery is unclear and further study is required.

Also, we assessed and correlated such changes with creatinine levels and recorded mean RIs of 0.73 and 0.86 in cases and controls, respectively, with a serum creatinine of 0.6-1.1 mg/dL, which was statistically significant. The mean RI levels in cases and controls with a serum creatinine of ≥1.2 mg/dL were 0.68 and 0.86, respectively, which was statistically significant. The impact of hypertension on vessels is well-established, and its impact on kidney function has been confirmed by many researchers over the years [22]. The elevated levels of serum creatinine are on par with those of severe hypertension, which may impact the RI directly or indirectly.

In our study of the evaluation of PI, we found the mean PIs in controls and cases to be 1.96 and 1.17, respectively (preeclampsia and eclampsia were 1.31 and 1.06, respectively), which was statistically significant. We also found a drop in the mean PI levels to be lower in the eclampsia cases than in the preeclampsia cases and the controls. Sahana et al. also reported a drop in the mean PI levels when the mean PIs of preeclampsia controls to cases were 1.35 and 1.05, respectively [18]. This result was confirmed by Kumari et al. who reported the mean PIs of the normotensive and preeclamptic participants to be 2.10 and 1.40, respectively [8]. 

A correlation of PI levels with platelets showed the mean PIs in cases and controls with platelets >150 x 10^9^/L to be 1.14 and 1.38, respectively, which was statistically significant. When the cases and controls had a serum creatinine level of 0.6-1.1 mg/dL, the mean PIs were 1.28 and 1.97, respectively, which was statistically significant. Similarly, the mean PIs in cases and controls with a serum creatinine of ≥1.2 mg/dL were 1.16 and 1.9, respectively, which was statistically significant. In all conditions, we observed a clear drop in the mean PI levels in cases compared to controls, more so in eclampsia cases than in preeclampsia cases.

We also evaluated the mean enveloped velocity in these patients and found the mean enveloped velocities in preeclampsia and eclampsia were 28.82 cm/sec and 20.43 cm/sec, respectively, which was also statistically significant. Our results were in accordance with other studies; however, they assessed the mean peak systolic velocity (PSV) only against the controls and not between preeclampsia and eclampsia cases. Sahana et al. [18] reported the mean PSVs of preeclampsia cases and controls to be 26.3 cm/sec and 38.0 cm/sec, respectively, and Kumari et al. [8] found the mean PSVs of preeclampsia cases and controls were 52.5 cm/sec and 56.90 cm/sec, respectively.

Therefore, we found a clear reduction in the RI, PI, and mean enveloped velocity levels in case subjects compared to controls, reflective of the impact of hypertension on the vessels. In our extensive study evaluating the various parameters, we found that the RI and PI in hypertensive pregnant women play a crucial role in determining the severity of damage to the eye, which may lead to partial or complete blindness if left unattended.

However, it may be difficult to establish a definite cause-effect relationship between these parameters with the small sample size based on a single medical center. Therefore, we advocate a multicentric, large sample, randomized-controlled, and standardized study to eliminate all the shortcomings encountered in our study.

## Conclusions

We found a definite drop in the PI, RI, and mean enveloped velocity levels in preeclampsia and eclampsia patients in comparison to those of normal subjects. Visual involvement is not uncommon in pregnancy-induced hypertension; however, these changes rarely lead to a loss of vision, resulting in permanent disability. Therefore, it is important to assess and manage such conditions at the earliest stage to achieve a better prognosis while providing adequate treatment to the patient for which the ophthalmic artery Doppler can serve as a useful tool for early detection of hemodynamic changes and ultimately help in better patient management. 
